# Prognostic Analysis for Patients With Parathyroid Carcinoma: A Population-Based Study

**DOI:** 10.3389/fnins.2022.784599

**Published:** 2022-02-18

**Authors:** Bei Qian, Ying Qian, Longqing Hu, Shoupeng Zhang, Li Mei, Xincai Qu

**Affiliations:** ^1^Department of Thyroid and Breast Surgery, Union Hospital, Tongji Medical College, Huazhong University of Science and Technology, Wuhan, China; ^2^Department of Pharmacy, Jingzhou Hospital, Yangtze University, Jingzhou, China

**Keywords:** parathyroid carcinoma, prognostic factor, cancer-specific survival (CSS), nomogram, validation

## Abstract

**Background:**

Parathyroid carcinoma (PC) is a rare but often lethal malignancy for which staging system, prognostic indicators, and treatment guidelines are still not established. We aimed to explore the prognostic parameters and construct a nomogram for cancer-specific survival (CSS) of PC.

**Methods:**

A retrospective analysis of 604 PC patients in the SEER database from 2001 through 2018 was performed. All the cases were randomly assigned to the training cohort (*n* = 424) or the validation cohort (*n* = 180) at a ratio of 7:3. The Kaplan–Meier method and Cox regression model were applied to estimate the CSS and risk factors, and a nomogram was constructed. The predictive accuracy and discriminative ability of the nomogram in CSS were assessed by concordance index (C-index), the area under the curve (AUC) of receiver operating characteristics (ROC), and the calibration curve.

**Results:**

Age at diagnosis > 70 years [hazard ratio (HR): 3.55, 95% CI: 1.07–11.78, *p* = 0.039] and tumor size > 35 mm (HR 4.22, 95% CI: 1.67–10.68, *p* = 0.002) were associated with worse CSS. Compared with distant metastasis, localized (HR 0.17, 95% CI: 0.06–0.47, *p* = 0.001) and regional lesions (HR 0.22, 95% CI: 0.07–0.66, *p* = 0.007) showed an improved CSS rate. Parathyroidectomy was the recommended treatment (*p* = 0.02). The C-index of the nomogram was 0.826, and the AUC for 5-, 10-, and 15-year CSS was 83.7%, 79.7%, and 80.7%, respectively. The calibration curve presented good agreement between prediction by nomogram and actual observation.

**Conclusion:**

Age at diagnosis > 70 years, tumor size > 35 mm, and distant metastasis were independent risk factors for PC-specific mortality. Parathyroidectomy was currently the most recommended treatment for PC. This nomogram provided individualized assessment and reliable prognostic prediction for patients with PC.

## Background

Parathyroid carcinoma (PC) is a rare endocrine malignancy, accounting for 0.005% of all malignancies (Salcuni et al., 2018) and 0.5–5% of all patients with primary hyperparathyroidism (PHPT) (Rawat et al., 2005). Patients with PC are often characterized by markedly elevated serum calcium and parathyroid hormone (PTH) (Cetani et al., 2016) who typically present with metabolic complications, including renal failure, bone disease, pancreatitis, cardiac arrhythmia, and occasionally a neck mass (Schulte and Talat, 2012). Most patients succumb to target organ damage caused by uncontrollable hypercalcemia rather than tumor burden. As PC is difficult to distinguish from parathyroid adenoma (PA), its preoperative and even intraoperative diagnoses are challenging. Preoperative ultrasound, although not suggestive of malignancy, is usually helpful in locating the abnormal parathyroid glands. As a supplement, CT may reveal an invasive parathyroid tumor and suggest a possible malignancy (Harari et al., 2011). However, it was reported that about 25% of cases are not being recognized by the surgeon at the time of initial parathyroidectomy (Kebebew, 2001). In terms of molecular diagnostics, if the PHPT patients were found to have the CDC73 (alternatively known as HRPT2) and/or MEN1 gene mutation, PC should be highly suspected (Schulte and Talat, 2012). The histological characteristics of PC reported in the literature include capsule infiltration, angioinvasion, tumor necrosis, fibrosis, numerous mitotic figures, and nuclear atypia (Schantz and Castleman, 1973; Bondeson et al., 1993). The use of adjuvant therapy is currently controversial owing to the lack of evidence-based clinical practice. Radical surgery with sufficient margins has been recommended as the only potential cure for PC (Schulte et al., 2014), although there is no consensus on a systematic oncological surgical approach or a dedicated terminology to describe it. Postoperatively, the clinical course of PC patients varies greatly. Recurrence was most common within the neck, with an r rate of 40–60% (Lee et al., 2007; Talat and Schulte, 2010). Distant metastasis occurs in about 30% of cases, usually to the lung and bone, and less frequently to the liver and visceral organs (Schulte and Talat, 2012; Lenschow et al., 2020).

Nevertheless, due to the rarity of PC and the paucity of large cohort studies or prospective research, it is still difficult to counsel patients on their natural course and prognosis. A population-based database allows us to have a large enough sample size to answer this question. In this study, we sought to conclude the clinicopathological features and explore the prognostic factors associated with cancer-specific survival (CSS) utilizing the national Surveillance, Epidemiology, and End Results (SEER) database. Meanwhile, we developed and internally validated a clinical nomogram incorporating independent prognostic factors, which can predict the CSS in patients with PC. It may provide individualized assessment and reliable prognostic prediction for patients with PC, which may build a foundation for a staging system.

## Materials and Methods

### Data Source and Patient Selection

The data of patients with PC from 2001 through 2018 were extracted from 18 population-based cancer registries of the SEER database^1^ using the SEER*Stat program (version 8.3.9), which is a cancer incidence registry that includes about 30% of the United States population. The extraction conditions were as follows: “Primary Site = C75.0-Parathyroid gland” and “Behavior code ICD-0-3 = Malignant.” The following variables were extracted: patient ID, Age at diagnosis, Sex, Year of diagnosis, Race/ethnicity, Laterality, Histology, Combined Summary Stage (extent of disease), Chemotherapy recode, Radiotherapy recode, Surgery of primary site, Tumor Size, Regional nodes examined, Regional nodes positive, Survival months, and SEER cause-specific death classification. The exclusion criteria in the study were as follows: a) unknown vital status (study cutoff used) and b) metastatic disease originating from other organ sites. The demographic and clinicopathological data of all eligible cases were collected and retrospectively analyzed.

### Cohort Definition and Variable Recode

The patients with PC were divided into the training and validation cohorts with a ratio of 7:3 using the R studio (version 4.0.3^2^) function “createDataPartition” to ensure that outcome events were distributed randomly between the two cohorts. The training cohort was used to screen variables and construct the nomogram predictive model, while the validation cohort was applied to validate the model based on the training cohort.

The variables from the selected cohorts included the following: age at diagnosis (≤70 and > 70), gender (male and female), race (white, black and other), extent of disease (distant, regional, localized, and unknown), surgery of primary site, radiotherapy (yes and no), lymph node (LN) metastasis (yes and no), and tumor size (≤35 and > 35 mm). The cutoff point of continuous variables such as age and tumor size for risk stratifications was generated by the “surv_cutpoint” function of the “Survminer” R package. Surgery of primary site was divided into four subgroups: no surgery, en bloc radical resection, parathyroidectomy (simple/partial surgical removal of primary site, total surgical removal of primary site and local tumor excision), and other.

The main endpoint was CSS according to data in the SEER database. CSS was defined as the proportion of patients with a type of cancer who did not die of cancer after a specific time period. These patients may still be alive, or they may have died of some other cause.

### Construction of the Nomogram

Cancer-specific survival of patients in the different risk groups was assessed using the Kaplan–Meier method. Univariate Cox proportional hazards model was used to check each factor’s power in predicting CSS. Subsequently, factors with a *p*-value < 0.05 in univariate analysis were further analyzed in a multivariate Cox proportional hazards model using a backward model selection procedure (elimination criterion: *p* > 0.10). The hazard ratios (HRs) and corresponding 95% CIs were calculated. Finally, according to the regression coefficients of each independent risk factor in the multivariate analysis, the nomogram was visualized to predict the probability of 5-, 10-, and 15-year CSS rates in patients with PC.

### Discrimination and Calibration of the Nomogram

The performance of the nomogram was evaluated by discrimination and calibration. The C-index was calculated to reflect the discrimination ability. The value of the C-index varies from 0.5 to 1.0, where 0.5 represents random chance, and 1.0 indicates a perfect ability to stratify patients into different prognosis groups. Meanwhile, time-dependent receiver operating characteristic (tROC) curves and the corresponding area under the curve (AUC) values at 5, 10, and 15 years were utilized to estimate the predictive accuracy. Typically, C-index and AUC values greater than 0.7 suggest a reasonable estimation.

Calibration curves with 1,000 bootstrap resamples were generated to test the calibration of the nomogram, which showed the correlation between the predicted probability and the frequency of the observed outcome. The standard curve was a straight line with slope 1 through the origin of the axes. The closer the calibration curve was to the standard curve, the better the prediction ability of the nomogram was.

### Statistical Analysis

Continuous variables conforming to the normal distribution were represented as the mean ± SD; otherwise, the median and interquartile range (IQR) were used. Categorical variables were shown as frequencies and their proportions. Statistical differences of distribution in variables between the training cohort and the validation cohort were analyzed by using the chi-square test. Statistical significance was cohort at two-sided *p* < 0.05. All statistical analyses and visualization are performed using the R studio version 4.0.3 software (see text footnote 2).

### Ethics Statement

This study was exempt from the approval processes of the Institutional Review Boards because the SEER database patient information was de-identified.

## Results

### Demographics and Clinicopathological Characteristics of Patients

**FIGURE 1:**
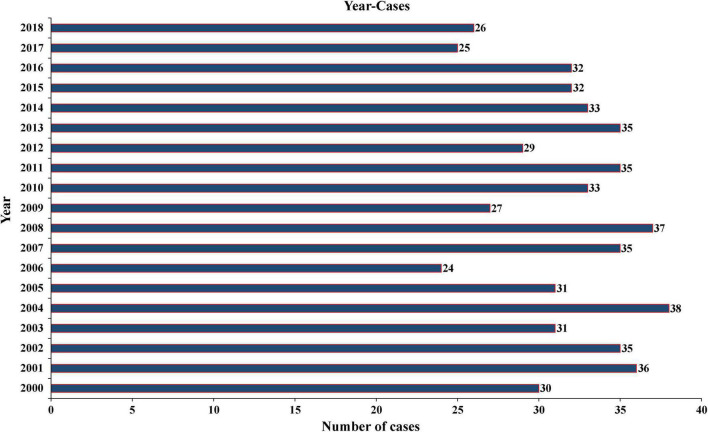
Annual number of PC cases recorded in 18 registries of the United States from 2001 to 2018. PC, parathyroid carcinoma.

A total of 604 patients with PC were extracted from the SEER database from 2001 to 2018. The baseline clinicopathological characteristics and treatment information of all patients were summarized in Table 1. The median age of all patients was 59 years (IQR: 48–68). The incidence of PC was roughly the same in men (51.5%) and women (48.5%). Among all patients, 455 (75.3%) patients were white, 601 (99.5%) patients had a unilateral lesion, 543 (89.9%) patients were not recorded to have a specific pathological type, and 577 (95.5%) patients accepted the treatment. A minority of patients who had distant metastasis (5.0%) and LN metastasis (6.1%) experienced radiotherapy (10.6%) and chemotherapy (0.2%). The median follow-up time was 76 months (IQR: 33–135), and the proportion of tumor-specific deaths was about 10.3%. The number of cases diagnosed each year was visualized as Figure 1, which showed that the incidence rate of PC presented a steady trend over the past two decades. The patient characteristics of the training (*n* = 424) and validation cohorts (*n* = 180) were concluded in Table 2. Since all cases were randomly assigned to the two cohorts, there was no statistical difference in the distribution of variables between them (all the *p*-values > 0.05).

**TABLE 1:** Demographics and clinicopathological characteristics of all patients with parathyroid carcinoma.

Characteristic	Level	Number/Median	%/IQR
*N*		604	100%
Age (year)		59	[48.0, 68.0]
Age group			
	≤20	4	0.7%
	>20, ≤40	77	12.7%
	>40, ≤60	255	42.2%
	>60, ≤80	232	38.4%
	>80	36	6.0%
**Gender**			
	Male	311	51.5%
	Female	293	48.5%
**Race**			
	White	455	75.3%
	Black	98	16.2%
	Other	51	8.4%
Follow-up time (months)		76	[33.0,135.0]
**Laterality**			
	Unilateral	601	99.5%
	Bilateral	3	0.5%
**Histology type**			
	Carcinoma, NOS	543	89.9%
	Papillary carcinoma	24	4.0%
	Adenocarcinoma	20	3.3%
	Other	17	2.8%
**Extent of disease**			
	regional	178	29.5
	localized	347	57.5%
	distant	30	5.0%
	unknown	49	8.1%
**Treatment**			
	Yes	577	95.5%
	No	27	4.5%
**Surgery**			
	Parathyroidectomy	488	80.8%
	En bloc radical resection	51	8.4%
	Other	38	6.3%
	No surgery	27	4.5%
**Radiotherapy**			
	Yes	64	10.6%
	No	540	89.4%
**Chemotherapy**			
	Yes	1	0.2%
	No	603	99.8%
**Lymph node metastasis**			
	Yes	37	6.1%
	No	135	22.4%
	NA	432	71.5%
**Tumor size (mm)**			
	≤20	125	20.7%
	>20, ≤40	189	31.3%
	>40, ≤60	43	7.1%
	>60	20	3.3%
	NA	227	37.6%
**Tumor-specific death**			
	Yes	62	10.3%
	No	542	89.7%

*IQR, interquartile range; NOS, not otherwise specified; mm, millimeter.*

**TABLE 2:** Baseline clinicopathological characteristics and treatment experience of patients in the training and validation cohort (the subdivision of the continuous variables age and diameter of the tumor generated by the “surv_cutpoint” function of the “Survminer” R package).

Characteristic	Level	Training cohort	Validation cohort	*P* value
*N*	604	424	180	
Age (%)				0.853
	≤70	335 (79.0)	141 (78.3)	
	>70	89 (21.0)	39 (21.7)	
Gender (%)				0.903
	Female	205 (48.3)	88 (48.9)	
	Male	219 (51.7)	92 (51.1)	
Race (%)				0.242
	White	314 (74.1)	141 (78.3)	
	Black	69 (16.3)	29 (16.1)	
	Other	41 (9.7)	10 (5.6)	
Region (%)				0.807
	unknown	36 (8.5)	13 (7.2)	
	regional	124 (29.2)	54 (30.0)	
	localized	245 (57.8)	102 (56.7)	
	distant	19 (4.5)	11 (6.1)	
Surgery (%)				0.780
	No surgery	21 (5.0)	6 (3.3)	
	En bloc radical resection	35 (8.3)	16 (8.9)	
	Other	28 (6.6)	10 (5.6)	
	Parathyroidectomy	340 (80.2)	148 (82.2)	
Radiotherapy (%)				0.549
	No	377 (88.9)	163 (90.6)	
	Yes	47 (11.1)	17 (9.4)	
Tumor size (%)				0.473
	≤35	192 (45.3)	89 (49.4)	
	>35	66 (15.6)	30 (16.7)	
	NA	166 (39.2)	61 (33.9)	
Lymph node metastasis (%)				0.458
	No	89 (21.0)	46 (5.6)	
	Yes	27 (6.4)	10 (5.6)	
	NA	308 (72.6)	124 (68.9)	
Tumor-specific death (%)				
	No	383 (90.3)	159 (88.3)	0.460
	Yes	41 (9.7)	21 (11.7)	

### Independent Prognostic Factors in the Training Cohort

**FIGURE 2:**
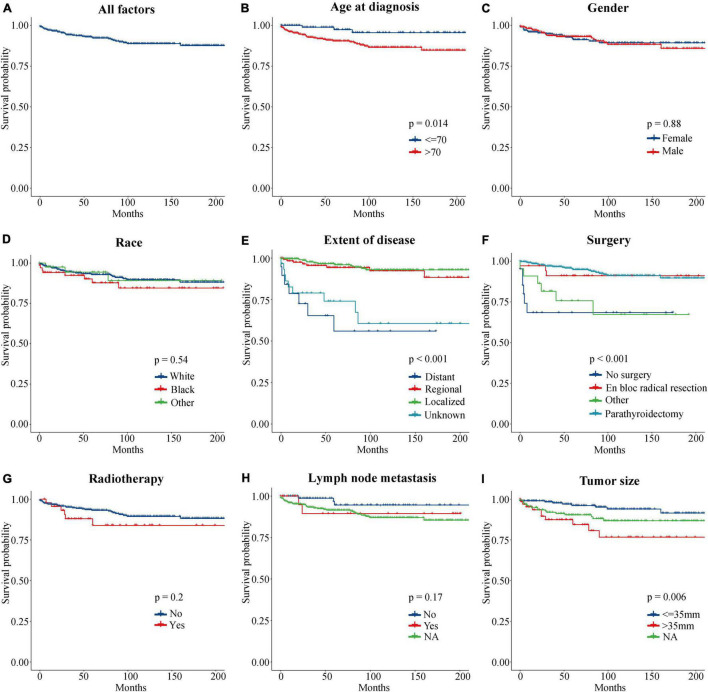
Kaplan–Meier curves of CSS for PC patients at different stages or with different risks. **(A)** All factors. **(B)** Age at diagnosis. **(C)** Gender. **(D)** Race. **(E)** Extent of disease. **(F)** Surgery. **(G)** Radiotherapy. **(H)** Lymph node metastasis. **(I)** Tumor size. CSS, cancer-specific survival; PC, parathyroid carcinoma.

**FIGURE 3:**
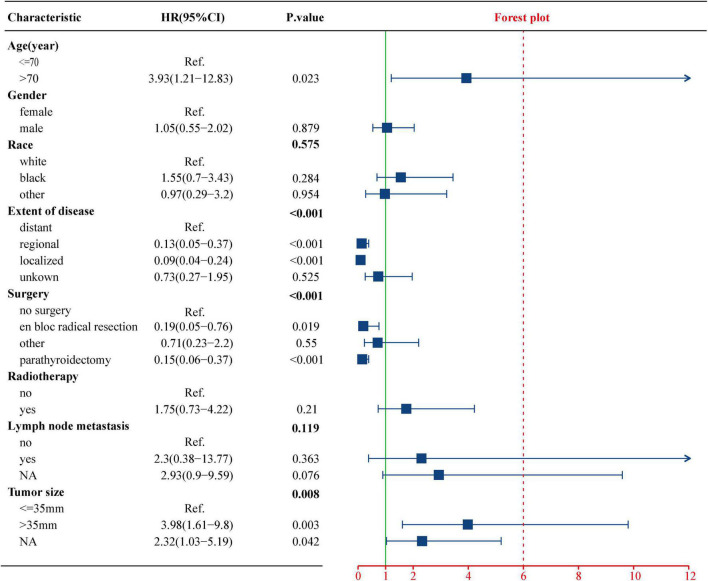
Univariate Cox regression analysis of the clinicopathological parameters for CSS using the training cohort. CSS, cancer-specific survival.

**FIGURE 4:**
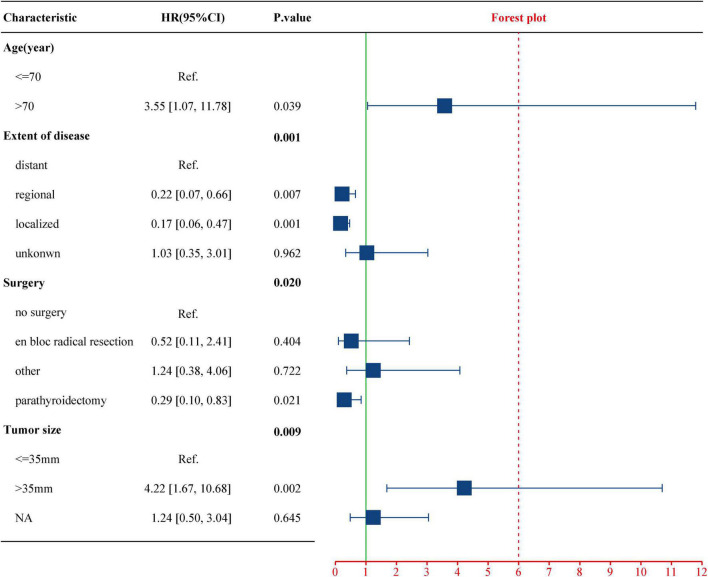
Multivariate Cox regression analysis of the select factors for CSS using the training cohort. CSS, cancer-specific survival.

To evaluate the impact of different factor on CSS of PC patients, the Kaplan–Meier survival analysis was performed in the training cohort. As shown in Figure 2, there were significant differences of CSS among age (*p* = 0.023), extent of disease (*p* < 0.001), surgery (*p* < 0.001), and tumor size (*p* = 0.008). Further, based on the univariate (Figure 3) and multivariate Cox (Figure 4) proportional hazards regression analyses, four independent prognostic factors were identified in the training cohort: age (>70: HR 3.55, 95% CI: 1.07–11.78, *p* = 0.039), tumor size (>35 mm: HR 4.22, 95% CI: 1.67–10.68, *p* = 0.002), extent of disease (localized: HR 0.17, 95% CI: 0.06–0.47, *p* = 0.001; and regional: HR 0.22, 95% CI: 0.07–0.66; *p* = 0.007), and surgery (parathyroidectomy: HR 0.29, 95% CI: 0.10–0.83; *p* = 0.021) were all significantly associated with CSS in patients with PC.

### Prognostic Nomogram for Cancer-Specific Survival

**FIGURE 5:**
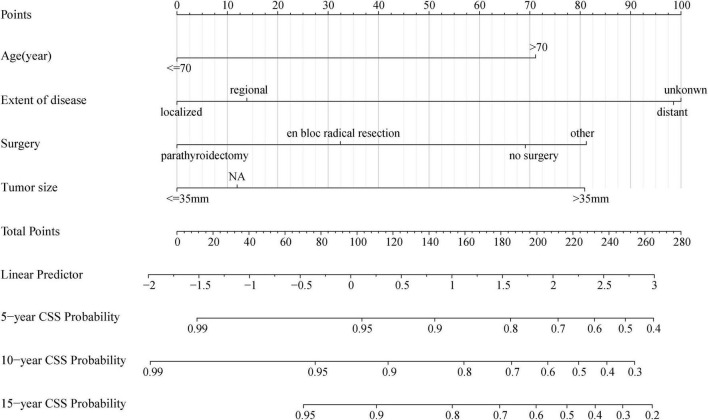
Nomogram for predicting probabilities of cancer-specific survival in patients with parathyroid carcinoma. NA, not availability.

As shown in Figure 5, the nomogram based on the above four independent prognostic factors was developed for the prediction of the 5-, 10-, and 15-year CSS rates in patients with PC. It demonstrated that the extent of disease contributed the most to the prognosis. Each level of each variable was assigned a score on the points scale. The total score was obtained by adding the scores of each selected variable. Then, the prediction corresponding to that total score helped to predict the CSS rate for each patient.

### Validation and Calibration of the Nomogram

**FIGURE 6:**
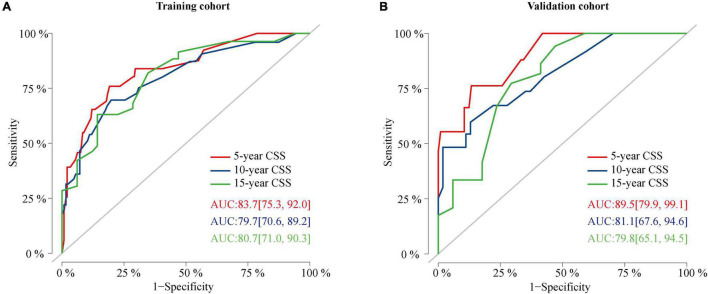
ROC curves of the nomogram. **(A)** ROC curves of the nomogram for predicting CSS probability at 5, 10, and 15 years in the training cohort. **(B)** ROC curves of the nomogram for predicting CSS probability at 5, 10, and 15 years in the validation cohort. ROC, receiver operating characteristic curve; CSS, cancer-specific survival.

**FIGURE 7:**
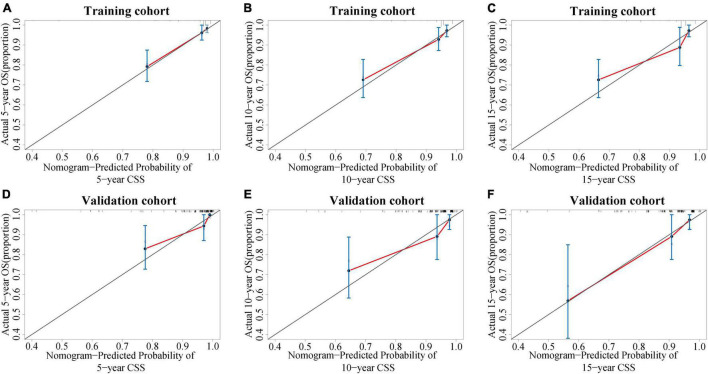
Calibration curves of the nomogram. **(A–C)** Calibration curves of the nomogram for predicting the 5-, 10-, and 15-year CSS of PC patients in the training cohort. **(D–F)** Calibration curves of the nomogram for predicting the 5-, 10-, and 15-year CSS of PC patients in the internal validation cohort. The x-axis indicates the predicted survival probability, and the y-axis indicates the actual survival probability. The diagonal 45° line (gray line) indicates that the prediction agreed with actuality. CSS, cancer-specific survival; PC, parathyroid carcinoma.

The C-index value was 0.826 in the training cohort and 0.872 in the validation cohort, which was greater than 0.7, reflecting the good discrimination ability of the model. The AUC values in the ROC curve were 83.7 (95% CI: 75.3–92.0), 79.7 (95% CI: 70.6–89.2), and 80.7 (95% CI: 71.0–90.3) in the training cohort and 89.5 (95% CI: 79.9–99.1), 81.1 (95% CI: 67.6–94.6), and 79.8 (95% CI: 65.1–94.5) at 5, 10, and 15 years in the validation cohort, respectively (Figure 6). The calibration curves also presented a favorable consistency between the actual observation and the nomogram prediction of the 5-, 10-, and 15-year CSS rates in both the training and validation cohorts (Figure 7).

## Discussion

The accurate and effective prognostic evaluation was of great clinical significance for individualized treatment and follow-up. However, due to the rarity of PC, there was little clinical evidence about its prognosis, and no predictive model was available for predicting the prognosis of patients with PC. In this population-based study, we identified four independent prognostic factors of the patients with PC based on the SEER database: age, extent of disease, surgical approach, and tumor size. At the same time, the nomogram prediction model was used to visualize the overall impact of these factors on the CSS rate of each patient. Validation of the nomogram showed it had good discriminative and calibration ability.

The incidence of PC was reported differently in different countries and regions. It was considered to account for approximately 1% of all cases of PHPT (Wilhelm et al., 2016) and 0.005% of all cancers in the United States (Ferraro et al., 2019). However, in China, the proportion of PC in the PHPT rose to 5–7% (Zhao et al., 2013), and its annual incidence in the European Union was about 2 cases per 10 million people in 2008 (van der Zwan et al., 2012). According to an analysis from the SEER database, the incidence of PC increased significantly from 3.58 to 5.73 per 10 million people from 1988 to 2003 (Lee et al., 2007). This may be due to the change in the National Institutes of Health (NIH) guidelines for parathyroidectomy for asymptomatic hyperparathyroidism (2002) leading to more PC patients being diagnosed early *via* increased serum calcium screening (Asare et al., 2015). Lo et al. (2018) reported that the increasing incidence was primarily due to smaller tumors (<3 cm) and regional disease (locally invasive and LN-positive disease). They also demonstrated that the incidence of PC has not increased since 2001 and instead remained stable, which was consistent with our findings. As shown in Figure 1, from 2001 to 2018, the number of cases recorded in 18 registered areas in the United States had remained at about 30 each year.

Regarding the effect of tumor size on the PC patient’s prognosis, previous studies have drawn different conclusions. Hundahl et al. (1999) analyzed 286 PC patients in the National Cancer Database (NCDB) from 1985 to 1995 and found that tumor size was not an important prognostic marker, which was supported by the research of Lee et al. (2007) and Talat and Schulte (2010). However, Hsu concluded that tumor size ≥ 3 cm was associated with LN metastasis (Hsu et al., 2014), and Asare et al. (2015) found that tumor size > 4 cm was correlated with an increased risk of death from PC. Among 520 patients with PC in the SEER database, tumor size > 3 cm was associated with worse CSS in patients with PC (HR 5.60, 95% CI: 1.50–21.20, *p* = 0.012) (Lo et al., 2018). A retrospective review from a tertiary-referral cancer hospital suggested that tumor size > 3.2 cm may increase the risk of distant metastasis by more than three times (Asare et al., 2019). In this study, Kaplan–Meier survival (Figure 2I) and Cox regression (Tables 1, 2) analysis jointly confirmed that tumor size > 35 mm was an independent adverse prognostic factor for PC patients. Patients with tumors size > 35 mm had an increased risk of cancer-specific death as compared to < 35 mm (HR 4.22, CI: 1.67–10.68, *p* = 0.002).

Distant metastasis was uniformly considered a poor prognostic factor for PC patients. In the present study, localized (HR 0.17, CI: 0.06–0.47, *p* = 0.001) or regional (HR 0.22, CI: 0.07–0.66, *p* = 0.007) PC showed significantly higher CCS as compared with distant metastases (in terms of the extent of disease, the SEER database defined “distant” as a neoplasm that had spread to parts of the body remote from the primary tumor either by direct extension or by discontinuous metastasis to distant organs or tissues or *via* the lymphatic system to distant LNs). Moreover, the nomogram exhibited that the degree of disease contributed the most to the prognosis (Figure 5). The primary data recorded for distant metastases in this study included the lung (4 cases), brain (1 case), bone (1 case), and other organs (2 cases). Similarly, Asare et al. (2019) found that distant metastasis reflected the highest risk factor for survival in patients with PC by compiling 37 years’ worth of patient management data from a single institution. At the same time, they noted that the lung was the most common site of metastasis and that patients with bone metastases may have a shorter survival period. Lee et al. (2007) and Harari et al. (2011) also clarified a definite association between metastatic disease at diagnosis and poorer overall survival (OS). Lo et al. (2018) confirmed that the presence of metastatic disease was prognostic and associated with CSS of PC patients.

Unexpectedly, no clear relationship was found between regional LN metastasis and prognosis of patients with PC (Figures 2H, 3). This may be due to the absence of complete information about LN status in the vast majority of patients (71.5%); no strong conclusion could be drawn about the prognostic value of LN status. The same phenomenon had been seen in similar studies of SEER databases (Hsu et al., 2014; Lo et al., 2018). However, some studies suggested that positive LNs were associated with poor prognosis. A study from the NCDB database confirmed that positive LNs predict lower OS and an increased risk of death (Sadler et al., 2014). The researchers suggested to remove ipsilateral central compartment LN dissection for patients with clinically suspicious LNs, but not prophylactic ipsilateral central LN dissection for all patients. And Schulte et al. (2012) proposed to place all PC patients with positive LNs, distant metastases, and vascular or tissue invasion into a high-risk category. Therefore, this study believed that the effect of LN status on the prognosis of PC patients still needed more prospective large-sample studies to confirm.

Age at diagnosis > 70 years proved to be an independent risk prognostic factor for PC, associated with poorer CSS (HR 3.55, 95% CI: 1.07–11.78, *p* = 0.039). Some previous studies had confirmed similar findings. Sadler et al. (2014) demonstrated that age at diagnosis > 57 years increased the risk of death 5 years after surgery and portended a poor OS. Lee et al. (2007) observed that young age was associated with an improved OS rate. However, since PC was a relatively indolent disease and the median age at diagnosis was 59 years (Table 1), OS as the primary endpoint may be confounded by lifespan and current comorbidity of the patients. Lo et al. had analyzed age at diagnosis as categorical variables, using thresholds of < 45, 45–59, 60–69, 70–79, and 80 + years, but did not find age at diagnosis to be associated with an increased risk of cancer-specific death. This may be due to the improper cutoff values concealing the difference between the age group. Silva-Figueroa et al. (2017) took the recurrence-free survival (RFS) as the endpoint of the study, confirming that age at diagnosis older than 65 years was negatively correlated with RFS rate (Zhou et al., 2021).

The only curative treatment of PC was surgery and the best chance of cure could be achieved by complete excision, avoiding capsular disruption at the first operation (Schulte and Talat, 2012; Wilhelm et al., 2016; Rodrigo et al., 2020). Survival analysis (Figure 2F) showed that the CSS rates of parathyroidectomy and en bloc radical resection were significantly higher than those of non-surgical patients (*p* < 0.001). Univariate Cox analysis showed that compared with non-surgical patients, parathyroidectomy (HR 0.15, CI: 0.06–0.37, *p* < 0.001) and en bloc radical resection (HR 0.19, CI: 0.05–0.76, *p* < 0.019) could improve the prognosis of PC patients, which was consistent with previous literature reports (Zhou et al., 2021). However, multivariate Cox analysis presented that only parathyroidectomy (HR 0.29, CI: 0.10–0.83, *p* = 0.021) was an independent prognostic factor for higher CSS rates. This finding could be interpreted as that the surgical treatment code in SEER was not specific enough to describe the exact scope of resection, which led to the possibility that the frequency of en bloc resection was underestimated. Regarding the management of the LNs, clinically suspected LNs should be removed, while prophylactic central or lateral neck dissection was not recommended (Christakis et al., 2016; Rodrigo et al., 2020).

For radiotherapy, neither survival analysis (*p* = 0.2) nor Cox regression analysis (*p* = 0.239) suggested that it could not improve the CSS rate in PC patients, which was consistent with the mainstream viewpoint (Asare et al., 2015; Christakis et al., 2016; Limberg et al., 2021). As PC was considered to be a radio-resistant tumor (Salcuni et al., 2018), a study by the Anderson Cancer Center confirmed that in high-risk cases, postoperative adjuvant radiotherapy achieves long-term disease control (Christakis et al., 2017). Therefore, the American Association of Endocrine Surgeons considered that adjuvant external radiation therapy should not be performed routinely after surgery but as a palliative option (Wilhelm et al., 2016). Chemotherapy was performed in only one patient in the study, and further analysis could not be explored. Nevertheless, literatures had certified that except for the partial response in a few case reports, cytotoxic chemotherapy has not shown effectiveness in the treatment of PC, and there were no standardized protocols to use it (Cetani et al., 2016; Tsoli et al., 2017). Additionally, this study also concluded that although there were multiple parathyroid glands, PC was mostly unilateral (99.5%), and the gender distribution for the 604 cases was nearly equal: 51.5% were male and 48.5% were female. There was no significant difference in the prognosis of PC among different races (*p* = 0.575).

As far as we know, this may be the first nomogram model to predict the CSS rate of patients with PC. Although the nomogram presented a good performance of discrimination and calibration, it has some limitations that should be acknowledged. Firstly, this study was a retrospective analysis with inherent biases. Secondly, the registry lacks preoperative calcium and PTH levels to identify biochemical prognostic. And recurrence was an important prognostic parameter for PC, but we were unable to evaluate it due to the lack of relevant records in the SEER database. Finally, although external validation was difficult to achieve due to the limited number of cases in a single institution, it was necessary.

## Conclusion

In conclusion, this study demonstrated a relatively stable incidence trend of PC over the past two decades. Age at diagnosis > 70 years, tumor size > 35 mm, and distant metastasis were independent risk factors for CSS in patients with PC. Gender, race, radiotherapy, and regional LN metastasis were not associated with CSS. Parathyroidectomy was currently the most recommended for PC. This nomogram provided individualized assessment and reliable prognostic prediction for patients with PC, which may build a foundation for a staging system. More future prospective studies are needed to confirm and improve this model.

## Data Availability Statement

Publicly available datasets were analyzed in this study. This data can be found here: https://seer.cancer.gov/data.

## Author Contributions

XQ: administrative support. BQ and YQ: collection and assembly of data. BQ, YQ, and LH: data analysis and interpretation. BQ: manuscript writing. SZ, LM, and XQ: critical revision of the manuscript. All authors contributed to the conception and design and approved the final manuscript.

## Conflict of Interest

The authors declare that the research was conducted in the absence of any commercial or financial relationships that could be construed as a potential conflict of interest.

## Publisher’s Note

All claims expressed in this article are solely those of the authors and do not necessarily represent those of their affiliated organizations, or those of the publisher, the editors and the reviewers. Any product that may be evaluated in this article, or claim that may be made by its manufacturer, is not guaranteed or endorsed by the publisher.

## Footnotes

1
https://seer.cancer.gov/

2
http://www.r-project.org

## References

[B1] Asare E. A. Silva-Figueroa A. Hess K. R. Busaidy N. Graham P. H. Grubbs E. G.. (2019). Risk of distant metastasis in parathyroid carcinoma and its effect on survival: a retrospective review from a high-volume center. *Ann. Surg. Oncol.* 26 3593–3599. 10.1245/s10434-019-07451-3 31111352

[B2] Asare E. A. Sturgeon C. Winchester D. J. Liu L. Palis B. Perrier N. D. (2015). Parathyroid carcinoma: an update on treatment outcomes and prognostic factors from the National Cancer Data Base (n.d.). *Ann. Surg. Oncol.* 22 3990–3995. 10.1245/s10434-015-4672-3 26077914

[B3] Bondeson L. Sandelin K. Grimelius L. (1993). Histopathological variables and DNA cytometry in parathyroid carcinoma. *Am. J. Surg. Pathol.* 17 820–829. 10.1097/00000478-199308000-00007 8338192

[B4] Cetani F. Pardi E. Marcocci C. (2016). Update on parathyroid carcinoma. *J. Endocrinol. Investig.* 39 595–606. 27001435 10.1007/s40618-016-0447-3

[B5] Christakis I. Silva A. M. Kwatampora L. J. Warneke C. L. Clarke C. N. Williams M. D. (2016). Oncologic progress for the treatment of parathyroid carcinoma is needed. *J. Surg. Oncol.* 114 708–713. 10.1002/jso.24407 27753088

[B6] Christakis I. Silva A. M. Williams M. D. Garden A. Grubbs E. G. Busaidy N. L. (2017). Postoperative local-regional radiation therapy in the treatment of parathyroid carcinoma: the MD Anderson experience of 35 years. *Pract. Radiat. Oncol.* 7 e463–e470. 10.1016/j.prro.2017.05.009 28751227

[B7] Ferraro V. Sgaramella L. I. Di Meo G. Prete F. P. Logoluso F. Minerva F. (2019). Current concepts in parathyroid carcinoma: a single centre experience. *BMC Endocr. Disord.* 19(Suppl. 1):46. 10.1186/s12902-019-0368-1 31142320 PMC6541564

[B8] Harari A. Waring A. Fernandez-Ranvier G. Hwang J. Suh I. Mitmaker E. (2011). Parathyroid Carcinoma: a 43-year outcome and survival analysis. *J. Clin. Endocrinol. Metab.* 96 3679–3686. 10.1210/jc.2011-1571 21937626

[B9] Hsu K. T. Sippel R. S. Chen H. Schneider D. F. (2014). Is central lymph node dissection necessary for parathyroid carcinoma? *Surgery* 156 1336–1341; discussion 41. 25456903 10.1016/j.surg.2014.08.005 PMC4254726

[B10] Hundahl S. A. Fleming I. D. Fremgen A. M. Menck H. R. (1999). Two hundred eighty-six cases of parathyroid carcinoma treated in the U.S. between 1985-1995: a National Cancer Data Base Report. The american college of surgeons commission on cancer and the american cancer society. *Cancer* 86 538–544. 10.1002/(sici)1097-0142(19990801)86:3<538::aid-cncr25>3.0.co;2-k 10430265

[B11] Kebebew E. (2001). Parathyroid carcinoma. *Curr. Treat. Options Oncol.* 2 347–354. 12057115 10.1007/s11864-001-0028-2

[B12] Lee P. K. Jarosek S. L. Virnig B. A. Evasovich M. Tuttle T. M. (2007). Trends in the incidence and treatment of parathyroid cancer in the United States. *Cancer* 109 1736–1741. 10.1002/cncr.22599 17372919

[B13] Lenschow C. Schrägle S. Kircher S. Lorenz K. Machens A. Dralle H. (2020). Clinical presentation, treatment, and outcome of parathyroid carcinoma: results of the NEKAR retrospective international multicenter study. *Ann. Surg.* 275 e479–e487. 10.1097/SLA.0000000000004144 32649472

[B14] Limberg J. Stefanova D. Ullmann T. M. Thiesmeyer J. W. Bains S. Beninato T. (2021). The use and benefit of adjuvant radiotherapy in parathyroid carcinoma: a national cancer database analysis. *Ann. Surg. Oncol.* 28 502–511. 10.1245/s10434-020-08825-8 32661850 PMC7357448

[B15] Lo W. M. Good M. L. Nilubol N. Perrier N. D. Patel D. T. (2018). Tumor size and presence of metastatic disease at diagnosis are associated with disease-specific survival in parathyroid carcinoma. *Ann. Surg. Oncol.* 25 2535–2540. 10.1245/s10434-018-6559-6 29971678 PMC8054302

[B16] Rawat N. Khetan N. Williams D. W. Baxter J. N. (2005). Parathyroid carcinoma. *Br. J. Surg.* 92 1345–1353. 16237743 10.1002/bjs.5182

[B17] Rodrigo J. P. Hernandez-Prera J. C. Randolph G. W. Zafereo M. E. Hartl D. M. Silver C. E. (2020). Parathyroid cancer: an update. *Cancer Treat. Rev.* 86:102012. 10.1016/j.ctrv.2020.102012 32247225

[B18] Sadler C. Gow K. W. Beierle E. A. Doski J. J. Langer M. Nuchtern J. G. (2014). Parathyroid carcinoma in more than 1,000 patients: a population-level analysis. *Surgery* 156 1622–1629; discussion 9–30. 10.1016/j.surg.2014.08.069 25456964

[B19] Salcuni A. S. Cetani F. Guarnieri V. Nicastro V. Romagnoli E. de Martino D. (2018). Parathyroid carcinoma. *Best Pract. Res. Clin. Endocrinol. Metab.* 32 877–889. 30551989 10.1016/j.beem.2018.11.002

[B20] Schantz A. Castleman B. (1973). Parathyroid carcinoma. A study of 70 cases. *Cancer* 31 600–605. 10.1002/1097-0142(197303)31:3<600::aid-cncr2820310316>3.0.co;2-0 4693587

[B21] Schulte K. M. Gill A. J. Barczynski M. Karakas E. Miyauchi A. Knoefel W. T. (2012). Classification of parathyroid cancer. *Ann. Surg. Oncol.* 19 2620–2628. 10.1245/s10434-012-2306-6 22434247

[B22] Schulte K. M. Talat N. (2012). Diagnosis and management of parathyroid cancer. *Nat. Rev. Endocrinol.* 8 612–622. 10.1038/nrendo.2012.102 22751344

[B23] Schulte K. M. Talat N. Galata G. Gilbert J. Miell J. Hofbauer L. C. (2014). Oncologic resection achieving r0 margins improves disease-free survival in parathyroid cancer. *Ann. Surg. Oncol.* 21 1891–1897. 10.1245/s10434-014-3530-z 24522991

[B24] Silva-Figueroa A. M. Hess K. R. Williams M. D. Clarke C. N. Christakis I. Graham P. H.. (2017). Prognostic scoring system to risk stratify parathyroid carcinoma. *J. Am. Coll. Surg.* 22 980–987. 10.1016/j.jamcollsurg.2017.01.060 28427885

[B25] Talat N. Schulte K. M. (2010). Clinical presentation, staging and long-term evolution of parathyroid cancer. *Ann. Surg. Oncol.* 17 2156–2174. 10.1245/s10434-010-1003-6 20221704

[B26] Tsoli M. Angelousi A. Rontogianni D. Stratakis C. Kaltsas G. (2017). Atypical manifestation of parathyroid carcinoma with late-onset distant metastases. *Endocrinol. Diabetes Metab. Case Rep.* 2017 17–106. 10.1530/EDM-17-0106 29118988 PMC5670324

[B27] van der Zwan J. M. Mallone S. van Dijk B. Bielska-Lasota M. Otter R. Foschi R. (2012). Carcinoma of endocrine organs: results of the RARECARE project. *Eur. J. Cancer* 48 1923–1931. 10.1016/j.ejca.2012.01.029 22361014

[B28] Wilhelm S. M. Wang T. S. Ruan D. T. Lee J. A. Asa S. L. Duh Q. Y. (2016). The american association of endocrine surgeons guidelines for definitive management of primary hyperparathyroidism. *JAMA Surg.* 151 959–968. 10.1001/jamasurg.2016.2310 27532368

[B29] Zhao L. Liu J. M. He X. Y. Zhao H. Y. Sun L. H. Tao B. (2013). The changing clinical patterns of primary hyperparathyroidism in Chinese patients: data from 2000 to 2010 in a single clinical center. *J. Clin. Endocrinol. Metab.* 98 721–728. 10.1210/jc.2012-2914 23365127

[B30] Zhou L. Huang Y. Zeng W. Chen S. Zhou W. Wang M. (2021). Surgical disparities of parathyroid carcinoma: long-term outcomes and deep excavation based on a large database. *J. Oncol.* 2021 1–8. 10.1155/2021/8898926 34135961 PMC8178016

